# A Trivalent HSV-2 gC2, gD2, gE2 Nucleoside-Modified mRNA-LNP Vaccine Provides Outstanding Protection in Mice against Genital and Non-Genital HSV-1 Infection, Comparable to the Same Antigens Derived from HSV-1

**DOI:** 10.3390/v15071483

**Published:** 2023-06-30

**Authors:** Kevin P. Egan, Sita Awasthi, Giulia Tebaldi, Lauren M. Hook, Alexis M. Naughton, Bernard T. Fowler, Mitchell Beattie, Mohamad-Gabriel Alameh, Drew Weissman, Gary H. Cohen, Harvey M. Friedman

**Affiliations:** 1 Infectious Disease Division, Department of Medicine, Perelman School of Medicine, University of Pennsylvania, Philadelphia, PA 19104, USA; kevinpe@pennmedicine.upenn.edu (K.P.E.); sawasthi@pennmedicine.upenn.edu (S.A.); giulia.tebaldi@pennmedicine.upenn.edu (G.T.); lhook@pennmedicine.upenn.edu (L.M.H.); alexis.naughton19@gmail.com (A.M.N.); tzel2395@gmail.com (B.T.F.); mg.alameh@pennmedicine.upenn.edu (M.-G.A.); dreww@pennmedicine.upenn.edu (D.W.); 2 Acuitas Therapeutics Inc., Vancouver, BC V6T 1Z3, Canada; mbeattie@acuitastx.com; 3 Department of Basic and Translational Sciences, School of Dental Medicine, University of Pennsylvania, Philadelphia, PA 19104, USA; ghc@upenn.edu

**Keywords:** HSV-1, HSV-2, HSV vaccine, nucleoside-modified mRNA, oral herpes, genital herpes, murine models, antibodies, T-cells, lipid nanoparticles

## Abstract

HSV-1 disease is a significant public health burden causing orofacial, genital, cornea, and brain infection. We previously reported that a trivalent HSV-2 gC2, gD2, gE2 nucleoside-modified mRNA-lipid nanoparticle (LNP) vaccine provides excellent protection against vaginal HSV-1 infection in mice. Here, we evaluated whether this HSV-2 gC2, gD2, gE2 vaccine is as effective as a similar HSV-1 mRNA LNP vaccine containing gC1, gD1, and gE1 in the murine lip and genital infection models. Mice were immunized twice with a total mRNA dose of 1 or 10 µg. The two vaccines produced comparable HSV-1 neutralizing antibody titers, and surprisingly, the HSV-2 vaccine stimulated more potent CD8^+^ T-cell responses to gE1 peptides than the HSV-1 vaccine. Both vaccines provided complete protection from clinical disease in the lip model, while in the genital model, both vaccines prevented death and genital disease, but the HSV-1 vaccine reduced day two vaginal titers slightly better at the 1 µg dose. Both vaccines prevented HSV-1 DNA from reaching the trigeminal or dorsal root ganglia to a similar extent. We conclude that the trivalent HSV-2 mRNA vaccine provides outstanding protection against HSV-1 challenge at two sites and may serve as a universal prophylactic vaccine for HSV-1 and HSV-2.

## 1. Introduction

Herpes simplex type 1 (HSV-1) infections affect an estimated 3.7 billion people between the ages of 0 and 49 worldwide [1]. Many people acquire HSV-1 during childhood from family members, friends, or peers. Orolabial HSV-1 infection is usually self-limited and although emotionally disturbing to some, does not pose a serious health risk to those infected. However, HSV-1 infection at other sites, such as the cornea or brain, may cause blindness [2] and life-threatening encephalitis [3], respectively. In many resource-rich countries, reduced orolabial HSV-1 acquisition during childhood has resulted in adolescents and adults having a higher risk of acquiring first-time HSV-1 as a genital infection [4]. Although genital HSV-1 infections have a reduced risk of recurrences [5,6], genital transmission of HSV-1 from mother to newborn during labor and delivery can be devastating. HSV-1 neonatal herpes is more common than HSV-2 neonatal herpes in resource-rich countries [7]. 

A prophylactic vaccine that prevents genital HSV-1 and HSV-2 infection and HSV-1 at non-genital sites is a public health priority. Despite major efforts from the scientific community, no FDA-approved vaccine is available to prevent HSV-1 or HSV-2 infection. Multiple observations from prior studies support continued efforts to develop an effective HSV-1 and HSV-2 vaccine. These include the following: (i) Mothers with recurrent genital herpes infections have a reduced risk of transmitting the infection to newborns compared to mothers with a first-time genital herpes infection, suggesting that the passive transfer of maternal antibodies from mother to fetus is protective [8]. (ii) Prior oral HSV-1 infection reduces the severity of subsequent genital HSV-2 infection [9]. (iii) Prior genital HSV-2 infection may provide complete protection against acquiring genital HSV-1 infection [10]. (iv) An HSV-2 glycoprotein D (gD2) subunit protein vaccine administered with monophosphoryl lipid A and alum protected HSV-1/HSV-2 double-seronegative women against HSV-1 genital disease [11]. Of interest, gD2 IgG binding antibodies, measured by ELISA, correlated with protection [12], and a subsequent analysis found that the gD2 subunit vaccine produced higher neutralizing antibody titers to HSV-1 than HSV-2 [13]. These data suggest that efforts to develop effective HSV vaccines are worthwhile and that an HSV-2 vaccine designed to prevent genital herpes may provide cross-protection against HSV-1 infection. 

We previously reported that a gC2, gD2, gE2 trivalent nucleoside-modified mRNA-lipid nanoparticle (LNP) vaccine, referred to here as tri-HSV-2, protected mice and guinea pigs against high-dose genital HSV-2 infection [14]. The tri-HSV-2 vaccine targets entry and immune evasion envelope glycoproteins on the HSV-2 virion that are also expressed at the infected cell surface. We noted that the tri-HSV-2 vaccine provided excellent protection against HSV-1 disease in mice [15]. When genital HSV-1 and HSV-2 infections were directly compared, the tri-HSV-2 immunization had greater efficacy against HSV-2 than HSV-1 infection. From these results, we hypothesized that an HSV-1 specific gC1, gD1, gE1 nucleoside-modified mRNA-LNP vaccine, referred to as tri-HSV-1, may provide better protection against HSV-1 than the tri-HSV-2 vaccine. This hypothesis is consistent with past vaccine efforts such as those for human papillomavirus that are type-specific with limited cross-protection against strains not included in the vaccine [16].

In this report, we generated a tri-HSV-1 mRNA vaccine and compared it to the tri-HSV-2 mRNA vaccine for efficacy against HSV-1 infection in mice. We challenged mice at two anatomical sites, the lip and the female genital tract. We found that the HSV-1 and HSV-2 vaccines were equally efficacious against HSV-1 infection in the lip and the immunizations were nearly equivalent in the genital infection model. We conclude that the tri-HSV-2 vaccine provides outstanding protection against HSV-1 infection at both oral and genital sites in mice.

## 2. Materials and Methods

### 2.1. Tri-HSV-1 and Tri-HSV-2 mRNA Immunogens

The production of tri-HSV-2 mRNA immunogens has been described previously [14]. The tri-HSV-1 immunogens were prepared the same way to generate mRNA that encodes the ectodomains of the gC1 (27–457) [17], gD1 (26–333) [18], and gE1 (24–309) [19] proteins, where the first amino acid listed represents the amino acid immediately after the signal sequence. Each mRNA contains the human IL-2 signal sequence replacing the endogenous viral signal sequence [20]. The three HSV-1 mRNAs were combined in equal concentrations based on mass prior to encapsulation in LNP by Acuitas. The vaccine formulations were stored at −80℃ and not reused once thawed.

### 2.2. Immunization of Mice

Female BALB/c mice aged 6–8 weeks old were immunized twice 30 days apart in the hind right gastrocnemius muscle [15]. Mice were immunized with either 1 µg or 10 µg (total) of tri-HSV-1 or tri-HSV-2 mRNA vaccines. Control mice were injected with an equal volume of sterile saline. Serum was collected 25 days after the final immunization and stored at −80 ℃.

### 2.3. Immunology Assays

(a) Serum IgG ELISA endpoint titers. Purified baculovirus-derived gC1, gD1, and gE1 were produced as previously described [17,18]. Purified protein was added to High Binding Costar microtiter plates [15]. Serial 2-fold dilutions of serum starting at 1:250 were added to protein-coated wells. Anti-mouse IgG was detected by adding 2,2′-azino-bis-3-ethylbenzothiazoline-6-sulfonic acid (ABTS). Endpoint titers were calculated as the serum concentration that had an absorbance value of ≥0.1 and was ≥two-fold higher than serum from PBS-immunized mice. 

(b) HSV-1 and HSV-2 neutralizing antibody titers. Neutralizing titers were measured by incubating 2-fold serial dilutions of mouse sera with 100 PFU of HSV-1 strain NS at 37 °C for 1 h. Incubations were carried out in the presence of 5% human serum as a source of complement obtained from an HSV-1/HSV-2 seronegative donor. Remaining infectious virus was determined by plaque assay on Vero cells. The neutralizing titer was defined as the highest serum dilution that reduced plaques by ≥50% [21].

(c) Isolation and stimulation of splenocytes from BALB/c mice. Mice were immunized twice with PBS or 10 µg of tri-HSV-1 or tri-HSV-2 vaccine. Immunized mice were sacrificed 10 days after the second immunization. The spleens were harvested, and mechanically dissociated into single-cell suspensions. Cell counts were obtained and 2 × 10^6^ cells were added to individual wells for each stimulation condition for each mouse. Splenocytes were stimulated in RPMI media supplemented with 55 µM 2-mercaptoethanol and 10 units of recombinant mouse IL-2/µL stimulation media. Intracellular cytokines were immobilized with 5 µg/mL Brefeldin A and Monensin. Cells were stimulated with dimethyl sulfoxide (DMSO), 0.04 µM phorbol-12-myristate 13-acetate (PMA) with 0.67 µM ionomycin, or 1 µg/mL of HSV-1 protein overlapping peptide pools. Cells were incubated for 12 h at 37 °C in 5% CO_2_. 

(d) Overlapping peptide pools. Overlapping peptide pools were synthesized by GenScript with a purity of ≥70%. Peptides for CD4^+^ and CD8^+^ T-cell stimulation were 15 amino acids long with an 11-amino-acid overlap. Identical amino acid sequences used in the mRNA immunogens were used for synthesizing overlapping peptide sequences. All peptide pools were resuspended in DMSO and RPMI to a concentration of 2 µg/µL and stored at −80 °C. 

(e) Flow cytometry reagents. All antibodies were purchased from BioLegend. Antibodies used for labeling cells were fluorescein isothiocyanate (FITC) conjugated anti-CD3 (clone 17A2), allophycocyanin (APC)-Fire 750 conjugated to CD4 (Clone GK1.5), phycoerythrin (PE) conjugated anti-IL-2 (clone JES6-5H4), PE-Dazzle 594 conjugated anti-CD107a (clone 1D4B), Peridinin-Chlorophyll-Protein-Cyanin (PerCP-Cy5.5) conjugated anti-CD8α (clone 53-6.7), Alexa-Fluor (AF)-700 conjugated anti-IFNγ (clone XMG1.2), Brilliant Violet 421 conjugated anti-IL-4 (Clone 11b11), and PE-Cy7 conjugated anti-TNFα (clone MP6-XT22). Dead cells were discriminated by Fixable Live-Dead Aqua dye (Invitrogen). Brefeldin A, PMA/ionomycin, Cyto-fast-fix-perm buffer, and intracellular staining permeabilization wash buffer were purchased from BioLegend. After labeling of intracellular cytokines, cells were fixed with 1% formaldehyde in PBS and stored at 4 °C until the next day. Cells were analyzed on a BD LSR 2. Gates were set to discriminate singlet cells and then lymphocytes based on forward scatter and side scatter properties. We collected 150,000 events in the lymphocyte gate. After data collection, flow samples were analyzed using FlowJo version 10.8 (Tree Star software). Cells were gated on CD4^+^ or CD8^+^ T-cells producing single cytokines. Boolean combination gates were used to compare IL-2, TNFα, and IFNγ production. 

### 2.4. Mouse Challenge Models

#### 2.4.1. HSV-1 Lip Infection

(a) Scratch inoculation of lips. Mice were challenged 30 days after the 2nd immunization. Mice were anesthetized with a solution of 100 mg/kg ketamine and 12.5 mg/kg xylazine, placed on their back, and a 25G needle was used to make 10 vertical scratches on the lower lip. After scratching the lip, 2 × 10^6^ PFU of HSV-1 strain NS was topically applied [22]. Mice were monitored for 13 days for weight loss and presence of lesions on the lower lip [23]. 

(b) HSV-1 isolation from lip and TG tissues. The lip and TG were removed and placed in tubes containing 500 µL DMEM supplemented with 5% FBS. The tissues were stored at −80 °C until further processing [23]. Lip and TG tissues were subjected to 3 freeze–thaw cycles. The lip was homogenized in a glass grinder and resuspended in 1 mL of media. The TG was homogenized using the frosted ends of glass slides and resuspended in 275 µL of DMEM. Tissue homogenates were used to make 10-fold serial dilutions that were then evaluated by plaque assay on Vero cells [23]. 

(c) HSV-1 DNA isolation from TG. The TG were harvested at the time of humane euthanasia or at the end of the experiment on day 28 post-challenge. Purified DNA was isolated and amplified for qPCR as previously described [15] and the HSV-1 DNA copy number was expressed as log_10_ DNA copies per 10^5^ adipsin genes. Samples with fewer than one copy by 40 cycles in duplicate wells were considered negative. If only one of the duplicate wells was positive, the sample was tested in triplicate and considered positive if two or more of the triplicates were positive.

#### 2.4.2. HSV-1 Vaginal Infection

(a) Mice were challenged with 2 × 10^6^ PFU of HSV-1 strain NS intravaginally [15]. Mice were scored daily for 14 days on the basis of one point each for redness/erythema, hair loss, distended belly indicating loss of fecal motility, and urinary staining for a maximum daily score of four. The vaginal canals of mice were swabbed on days 2 and 4 for virus titers. Vaginal swabs were placed in 1 mL of DMEM supplemented with L-glutamine, HEPES, antibiotics, and 5% FBS. Serial 10-fold dilutions were evaluated by plaque assay on Vero cells [15]. 

(b) HSV-1 DNA isolation from DRG. The DRG were harvested at the time of humane euthanasia or at the end of the experiment on day 28 post-challenge as described above for TG. 

### 2.5. Statistical Analysis

The log-rank test was used to calculate *p* values for survival curves. The Mann–Whitney test was used to calculate *p* values in experiments evaluating vaginal titers, serum antibodies by ELISA, HSV-1 neutralizing titers, viral titers present in lip and TG tissue, and HSV-1 DNA copy number in the lip, TG, and DRG. The flow cytometry data were analyzed using 2-way ANOVA with correction for multiple comparisons. All significance tests were two-tailed with a *p* value < 0.05 considered significant. Analysis was conducted using GraphPad Prism version 9.5 (GraphPad software Inc., San Diego, CA, USA).

## 3. Results

Our approach was to evaluate HSV-1 antibody and T-cell responses produced by the tri-HSV-1 and tri-HSV-2 vaccines, and then to assess the efficacy of the two vaccines in protecting against HSV-1 challenge in the mouse lip and mouse vaginal infection models. 

### 3.1. IgG ELISA and Neutralizing Antibody Responses to Tri-HSV-1 and Tri-HSV-2 Vaccines

**Figure 1:**
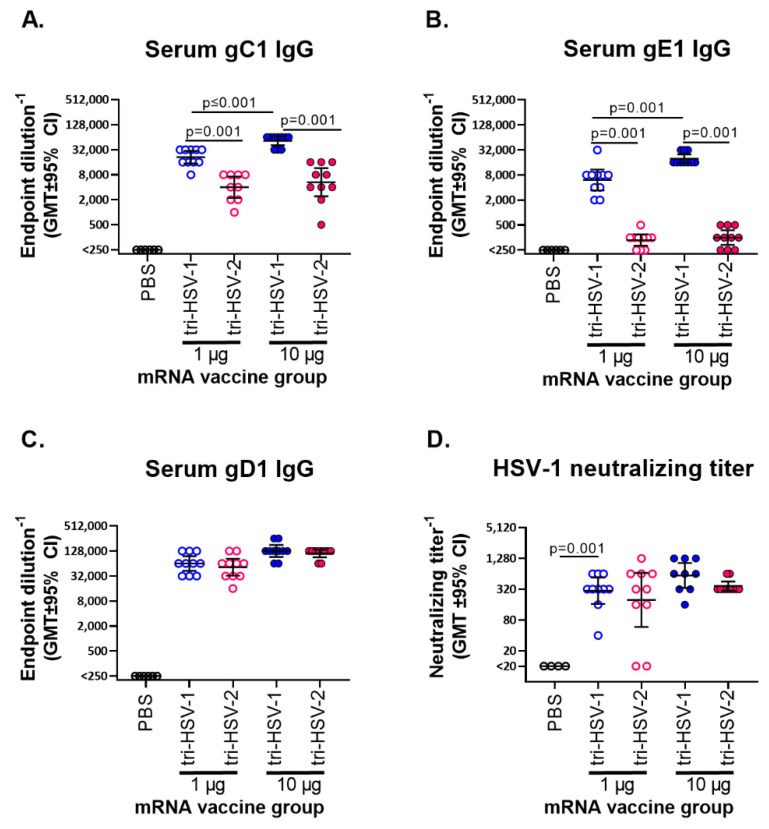
Tri-HSV-1 and tri-HSV-2 immunizations generate antibodies that bind to HSV-1 proteins and neutralize HSV-1. Mice were immunized twice with PBS, tri-HSV-1, or tri-HSV-2 mRNA at 1 µg or 10 µg of total mRNA. (**A**–**C**) IgG ELISA endpoint titers against HSV-1 proteins. (**D**) HSV-1 neutralizing titers in the presence of 5% human serum as source of complement. Experiments were performed once with 10 mice per group. *p* values were calculated using the two-tailed Mann–Whitney test. The blue circles represent the tri-HSV-1 vaccine and the red circles the tri-HSV-2 vaccine.

We immunized mice with either a 1 µg (low) or 10 µg (high) dose of total mRNA containing equal concentrations of each mRNA immunogen. We used a low and high vaccine dose to better resolve potential differences between the tri-HSV-1 and tri-HSV-2 vaccines. The tri-HSV-1-immunized mice had significantly higher serum IgG ELISA antibodies to gC1 and gE1 than tri-HSV-2-immunized animals (Figure 1A,B). The gC1 IgG levels were 4.6-fold higher in the tri-HSV-1 group than tri-HSV-2 at 1 µg and 6.9-fold higher at 10 µg doses. We detected a dose response with the tri-HSV-1 vaccine in that the gC1 IgG levels were 2.3-fold higher in animals immunized with 10 µg than 1 µg, while no differences were detected at the two doses in the tri-HSV-2-immunized mice (Figure 1A). The gE1 IgG levels were 35- and 72-fold higher in tri-HSV-1 than tri-HSV-2 immunized mice at the low and high doses, respectively. We detected a 2.5-fold dose–response increase in gE1 IgG titers in the high dose tri-HSV-1 immunized mice (Figure 1B). The gD1 IgG levels were not significantly different comparing tri-HSV-1- and tri-HSV-2-immunized mice at the low or high dose (Figure 1C). The IgG ELISA results suggest that many epitopes on gD1 and gD2 are type-common, while epitopes on gC1 and gC2 or gE1 and gE2 are type-specific.

We evaluated the sera for HSV-1 neutralizing antibodies in the presence of 5% human serum as a source of complement. Neutralizing titers were higher in the tri-HSV-1 mice at both immunization doses, but differences did not reach statistical significance (Figure 1D). The neutralizing titers are consistent with the observation that gD antibodies are more potent at neutralizing virus than gC or gE antibodies [19,21].

### 3.2. CD4^+^ and CD8^+^ T-Cell Responses Produced by Tri-HSV-1 and Tri-HSV-2 Immunization

**Figure 2:**
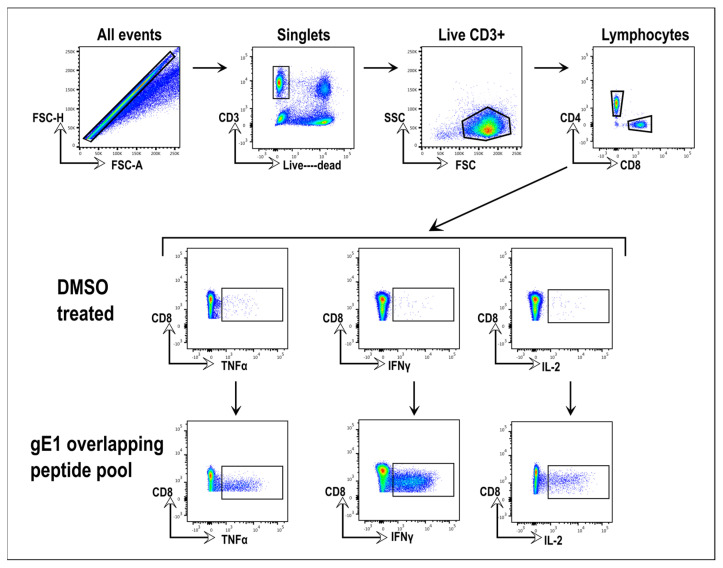
Gating strategy used in CD4 and CD8 T-cell flow cytometry analysis. Top row: SingleT-cells were gated, then live CD3^+^, then lymphocytes, and then CD4^+^ and CD8^+^ T-cells. Middle row: DMSO was the vehicle used to dissolve the overlapping peptide pools and was included as a negative control for the stimulation of cytokines in CD4^+^ and CD8^+^ T-cells in the absence of overlapping peptides. Single cytokine positive CD8^+^ T-cells were gated using CD8^+^ and TNFα, IFNγ, or IL-2 parameters. Bottom row: Gate coordinates were set based on the DMSO-treated cells shown in the middle row and those gate coordinates were transferred to the peptide-treated cells. An example is shown of TNFα, IFNγ, and IL-2 production by CD8^+^ cells of a mouse immunized with tri-HSV-2.

We next evaluated the T-cell response in mice immunized twice with 10 µg tri-HSV-1 or tri-HSV-2 vaccines. Splenocytes were stimulated with overlapping peptide pools of gC1, gD1, or gE1. Responding cells were measured by intracellular cytokine flow cytometry. The gating strategy is shown in Figure 2. 

**Figure 3:**
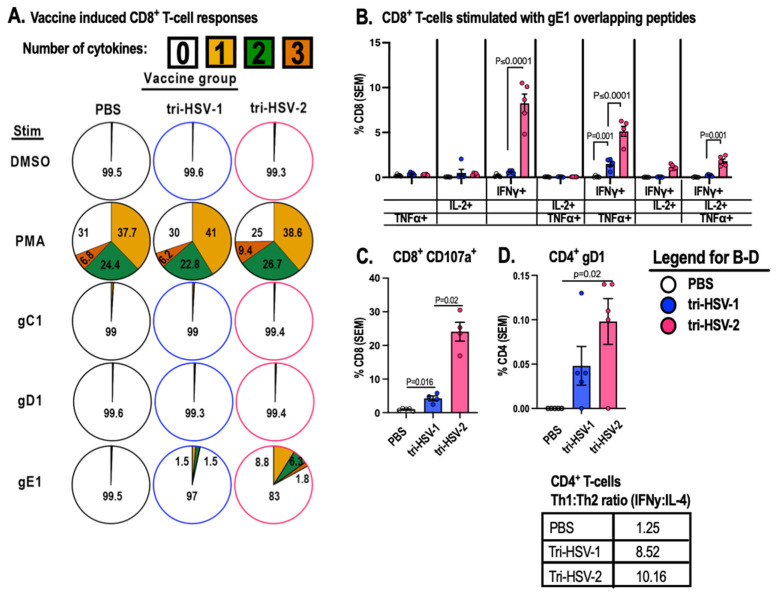
Tri-HSV-1 and tri-HSV-2 immunizations generate CD4^+^ and CD8^+^ T-cell responses to HSV-1-overlapping peptides. Mice were immunized twice with 10 µg total of tri-HSV-1, tri-HSV-2 mRNA, or PBS. (**A**) Proportion of CD8+ T-cells producing 0, 1, 2, or 3 cytokines. Numbers inside or adjacent to colored segments indicate percentage of total CD8^+^ T-cells producing the indicated number of cytokines. (**B**) Activation markers of CD8^+^ T-cells stimulated with gE1-overlapping peptide pool producing TNFα, IFNγ, and/or IL-2. (**C**) Proportion of CD8^+^ T-cells expressing CD107a when stimulated with gE1-overlapping peptides. (**D**) Proportion of CD4^+^ T-cells producing polyfunctional cytokines TNFα, IFNγ, and IL-2 following stimulation with a gD1-overlapping peptide pool. (**D table**) Ratio of CD4^+^ T-cells producing IFNγ to IL-4 following stimulation with gD1-overlapping peptides. Experiments were performed once with 5 mice per group for (**A**–**D**). *p* values in (**B**) were calculated using 2-way ANOVA with Tukey’s correction for multiple comparisons. *p* values in (**C**,**D**) were calculated using the two-tailed Mann–Whitney test.

We applied this gating strategy to measure IFNγ, TNFα, and IL2 production from CD4^+^ and CD8^+^ T-cells (Figure 3). We did not detect CD8^+^ T-cell responses to gC1 or gD1 in tri-HSV-1- or tri-HSV-2-immunized mice (Figure 3A). However, we did detect robust CD8^+^ T-cell responses to gE1-overlapping peptides, particularly in mice immunized with the tri-HSV-2 vaccine (Figure 3A). The number of IFNγ^+^TNFα^+^ (double-positive) CD8^+^ T-cells was 3.4-fold higher in the tri-HSV-2 immunized mice than the tri-HSV-1 group (Figure 3B, third group from right). Additionally, the number of triple-positive CD8^+^ T-cells (IFNγ^+^TNFα^+^IL-2^+^) was 8.2-fold higher in the tri-HSV-2 group compared to tri-HSV-1 (Figure 3B, last group on right). We also analyzed CD8^+^ T-cell activity by labeling with CD107a, a marker of degranulation [24]. The tri-HSV-1 group had more activated T-cells compared to the PBS group, but the tri-HSV-2 animals had 7-fold greater CD107a labeling compared to the tri-HSV-1 animals (Figure 3C). We next analyzed the CD4^+^ T-cells stimulated with gD1-overlapping peptides. CD4^+^ T-cells in the tri-HSV-2-immunized group had a 2-fold increase in polyfunctional cells (IFNγ^+^TNFα^+^IL-2^+^) compared to tri-HSV-1 mice (Figure 3D). We evaluated whether responding cells have a Th1 or Th2 phenotype by calculating the ratio of IFNγ^+^ (Th1) to IL-4^+^ (Th2) responses. We found a high ratio of IFNγ to IL-4, indicating a Th1 phenotype (Table under Figure 3D). We did not detect CD4^+^ T-cell responses to gC1- or gE1-overlapping peptides in tri-HSV-1- or tri-HSV-2-immunized mice. These results indicate that immunization with tri-HSV-2 mRNA induces CD4^+^ and CD8^+^ effector T-cell responses in mice to peptides from HSV-1, and that some CD4^+^ and CD8^+^ T-cell responses to the tri-HSV-2 vaccine are even more potent than to the tri-HSV-1 vaccine.

### 3.3. HSV-1 Challenge Infection Using the Mouse Lip Infection Model

**Figure 4:**
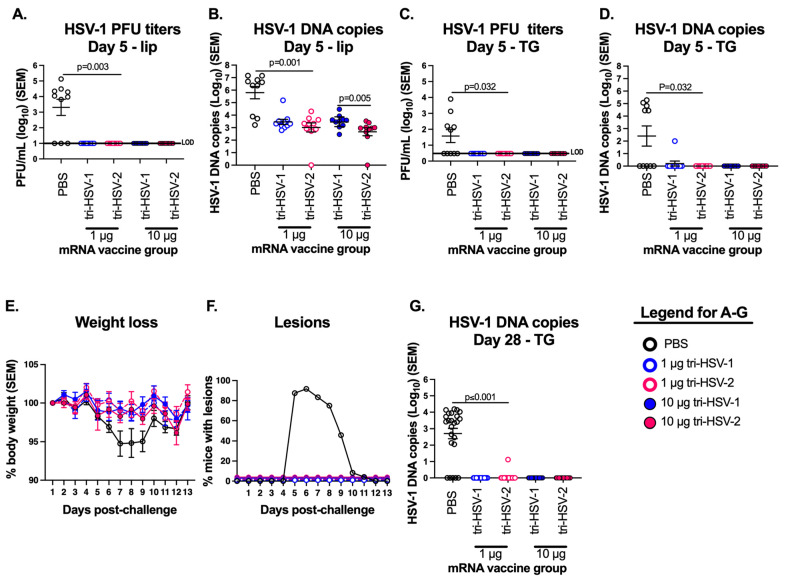
Tri-HSV-1 and tri-HSV-2 mRNA vaccines protect mice against acute and latent HSV-1 infection in the lip. Mice were immunized with PBS, tri-HSV-1, or tri-HSV-2 mRNA vaccines as in Figure 1. Mice were challenged with 2 × 10^6^ PFU of HSV-1 applied to the scarified lower lip. (**A**) HSV-1 virus titers in the lower lip. (**B**) HSV-1 DNA copy number in the lower lip. (**C**) HSV-1 virus titers in the trigeminal ganglia (TG). (**D**) HSV-1 DNA copy number in the TG. (**A**–**D**) Experiments were performed once with 10 mice per group. (**E**) Mice were weighed daily for 13 days. (**F**) Mice were scored for lesions on the lip with a score of 1 for lesions or 0 for no lesions. (**G**) HSV-1 DNA genome copies in the TG 28 days post challenge. (**E**–**G**) Experiments represent the pooled results of two experiments with 24 mice in the PBS group, and 15 mice in the tri-HSV-1 and tri-HSV-2 immunization groups. *p* values in (**A**–**D**,**G**) were calculated by the two-tailed Mann–Whitney test. LOD, limit of detection.

We selected a challenge model that targets a non-genital site for infection to compare the efficacy of tri-HSV-1 and tri-HSV-2 mRNA immunizations. The most common site of HSV-1 infection is in the oral mucosa. The lip scarification model allows us to add virus to the lower lip adjacent to the oral mucosa [23]. Scratch inoculation of the virus on the lower lip causes productive infection in the lip and trigeminal ganglia (TG) infection via HSV-1 transport along the mandibular branch of the trigeminal nerve. We immunized mice and later infected them by scratch inoculation with 2 × 10^6^ PFU of HSV-1-strain NS to the lower lip. PBS-immunized mice had high levels of replication competent virus in the lip, indicating productive infection; however, we did not detect replication-competent virus in the lip from any immunized mouse (Figure 4A). By qPCR, the PBS mice had an average 5.8 log_10_ of HSV-1 DNA present in the lip. In the mRNA groups, the 10 µg tri-HSV-1 group had the highest burden of HSV-1 DNA with an average 3.5 log_10_ of HSV-1 DNA. Although HSV-1 DNA was detected in the lip of mRNA-vaccinated animals, no replication-competent virus was recovered. The 10 µg tri-HSV-2 mRNA group had a significantly lower copy number of viral DNA compared to the 10 µg tri-HSV-1 group (Figure 4B). We next measured infectious virus in the TG 5 days post challenge. Five of ten (50%) PBS mice had replication-competent virus present in the TG, while no immunized mouse had replication-competent virus (Figure 4C). The presence of replication competent virus or HSV-1 DNA in the TG indicates a risk of virus establishing a latent infection. The same five of ten (50%) mice in the PBS group had viral DNA in the TG at 5 days (Figure 4D). Only 1/40 (2.5%) of the tri-HSV-1- or tri-HSV-2-immunized mice was positive for viral DNA present in the TG at 5 days. The single positive animal was in the 1 µg group of tri-HSV-1 (Figure 4D). 

We monitored mice for weight loss and lip lesions over 13 days. The PBS-vaccinated mice lost approximately 5% body weight between days 6 and 9 post infection. After day 10, the weight in the PBS mice recovered (Figure 4E). None of the immunized mice lost more than 3% body weight (Figure 4E). The PBS mice developed lesions on the lower lip 5 days post infection. At 6 days, 22/24 (92%) of PBS mice had lower lip lesions. The lesions healed over the next 4 days and were no longer present by day 11 (Figure 4F). None of the immunized mice developed lip lesions (Figure 4F). We analyzed the TGs for HSV-1 DNA copy number 28 days post-challenge as an indicator of latent HSV-1 infection. We detected HSV-1 DNA in the TG from 79% of the PBS mice (Figure 4G). Only a single vaccinated mouse in the 1 µg dose tri-HSV-2 group had HSV-1 DNA detected in the TG. The mean copy number of HSV-1 DNA in PBS mice was 2.7 log_10_, whereas the HSV-1 DNA copy number in the single breakthrough case in the tri-HSV-2-immunized mouse was 1.1 log_10_, a 2.5-fold reduction. These data suggest that the tri-HSV-1 and tri-HSV-2 mRNA vaccines provide excellent protection against high-dose HSV-1 challenge in the lip. Both vaccines were nearly perfect in preventing HSV-1 DNA from reaching the TG at acute and latent time points. The tri-HSV-1 and tri-HSV-2 vaccines had equivalent efficacy, even at a low dose of 1 µg total mRNA.

### 3.4. HSV-1 Challenge Infection Using the Mouse Genital Infection Model

**Figure 5:**
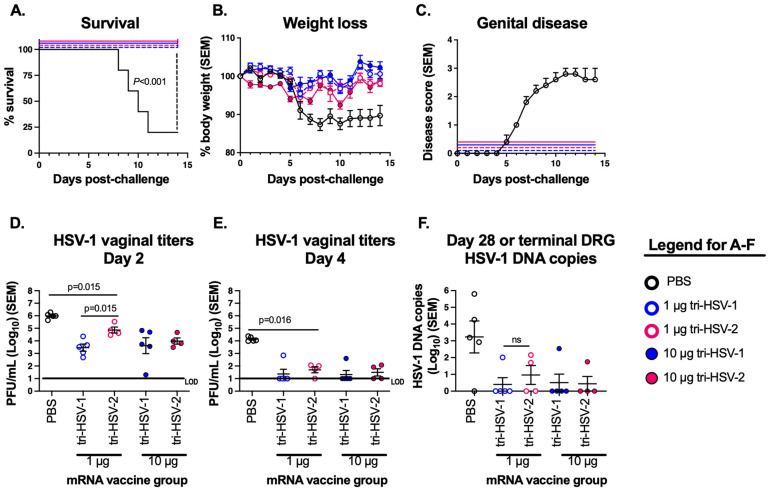
Tri-HSV-1 and tri-HSV-2 mRNA vaccines protect mice from intravaginal HSV-1 disease. Mice were immunized with PBS, tri-HSV-1, or tri-HSV-2 vaccines as in Figure 1. Mice were challenged with 2 × 10^6^ PFU of HSV-1 intravaginally and scored for clinical disease for 14 days. (**A**) Survival curve. *p* values were calculated by the log-rank test. (**B**) Weight loss. (**C**) Mean genital disease scores for each group. (**D**–**E**) Vaginal swabs were obtained on days two and four for virus titers. (**F**) HSV-1 DNA copy number in DRG. Experiments were performed once with 5 mice in the PBS group, 5 mice in the tri-HSV-1 group, and 4 mice in the tri-HSV-2 group. *p* values in (**D**–**F**) were calculated using the two-tailed Mann–Whitney test without adjustments for multiple comparisons.

We detected only minor differences in efficacy between tri-HSV-1 and tri-HSV-2 vaccines in the HSV-1 lip model. In our prior publication, we noted that 16/29 (55%) mice immunized with tri-HSV-2 had positive day 2 vaginal virus titers (mean titer 1.3 PFU/mL) after HSV-1 intravaginal challenge [15]. With room for improvement, we evaluated whether the tri-HSV-1 vaccine was better than the tri-HSV-2 vaccine in reducing day 2 or day 4 vaginal virus titers after HSV-1 challenge. We vaccinated mice with tri-HSV-1 and tri-HSV-2 mRNA vaccines at low (1 µg) and high (10 µg) doses, treated mice with Depo-Provera five days before infection, and challenged them with 2 × 10^6^ PFU of HSV-1-strain NS. The PBS-immunized animals started succumbing to infection by day 8. By day 14, only 1 of 5 (20%) survived the HSV-1 challenge, while none of the tri-HSV-1 or tri-HSV-2 mRNA-vaccinated mice died at 1 µg or 10 µg doses (Figure 5A). In the PBS group, mice started to lose weight by 6 days post-challenge, while the tri-HSV-1 and tri-HSV-2 immunized mice had little or no weight loss (Figure 5B). Mice were scored for genital disease over 14 days. The PBS-vaccinated mice developed genital disease on day 5 post challenge, which peaked on day 11 with a mean disease score of 2.8. We did not observe genital disease in any vaccinated mouse (Figure 5C). 

We next evaluated whether infectious virus was present in vaginal swabs obtained on days 2 or 4 post-challenge. We detected high titers of HSV-1 in all the PBS animals, with a mean titer of 10^6^ PFU/mL. Virus titers were significantly lower in all vaccine groups on days 2 (Figure 5D) and 4 (Figure 5E). Importantly, day 2 virus titers for the 1 µg tri-HSV-1 group were approximately 2 log_10_ lower than the 1 µg tri-HSV-2 group. This difference was the only statistically significant difference we detected favoring the tri-HSV-1 vaccine in this study and was limited to the 1 µg dose, while at the 10 µg dose in the lip model we noted the opposite result: A lower HSV-1 DNA copy number in the lips of tri-HSV-2 mice than tri-HSV-1 mice (Figure 4B and Figure 5D). We observed significant reductions in viral titers in all vaccine groups compared to PBS at 4 days post challenge with no difference between the tri-HSV-1 and tri-HSV2 mRNA groups (Figure 5E). We evaluated the HSV-1 DNA copy number present in the DRG at the time of humane sacrifice (PBS group) or 28 days post challenge. We detected high levels of HSV-1 DNA in the four PBS animals that were humanly euthanized, while the one surviving animal did not have HSV-1 DNA detected in the DRG at the end of the experiment (Figure 5F). We found no significant differences comparing the tri-HSV-1 and tri-HSV-2 groups. These results demonstrate that both the tri-HSV-1 and tri-HSV-2 vaccines protected mice against a high-dose HSV-1 intravaginal challenge, with only a very small difference favoring the tri-HSV-1 group for day 2 vaginal virus titers at the 1 µg dose.

## 4. Discussion

An effective prophylactic vaccine is the best approach to limit new genital and non-genital HSV infections. We previously reported that tri-HSV-2 mRNA immunization provides excellent protection in mice and guinea pigs against HSV-2 intravaginal challenge [14]. Tri-HSV-2 immunization stimulates robust CD4^+^ T-follicular helper cells and germinal center B cell responses [25], and generates high levels of neutralizing antibodies and antibodies that block gC2 binding to C3b and gE2 binding to the IgG Fc domain [26]. The gC2, gD2, and gE2 amino acid sequences share 65%, 82%, and 73% identity with gC1, gD1, and gE1, respectively. We evaluated whether this level of homology generates immune responses capable of potent cross-protection against HSV-1 infection at non-genital sites in mice and whether we can improve upon the protection against genital HSV-1 infection in mice using a tri-HSV-1 vaccine. 

The most common site of HSV-1 infection is orolabial. In contrast, HSV-2 rarely infects the oral cavity, suggesting that HSV-1 has a fitness advantage at this site. We chose to use the lip scarification model of HSV-1 infection to compare the immunization efficacy of the tri-HSV-2 vaccine with a tri-HSV-1 vaccine [23,27]. In this model, the epithelial surface was scratched to allow the virus to infect the underlying susceptible cells [28]. The tri-HSV-2 immunization provided total protection against clinical and subclinical infection. Importantly tri-HSV-2 mice were completely protected against HSV-1 DNA reaching the TG, indicating a minimal risk of latent infection and possible reactivation.

In our prior study, we noted excellent protection by the tri-HSV-2 vaccine against HSV-1 intravaginal infection in mice based on complete protection against death and clinical disease after a high-dose challenge; however, there was room for improvement based on day 2 and day 4 vaginal virus titers post challenge [15]. Therefore, we assessed whether the tri-HSV-1 vaccine provides better protection than the tri-HSV-2 vaccine in the murine genital infection model, particularly against subclinical infection measured by day 2 and day 4 vaginal virus titers. We noted a difference in vaginal titers comparing the 1 μg dose of tri-HSV-1 with 1 μg tri-HSV-2 on day 2 but no other difference. Our overall conclusion based on studies in mice is that there is no advantage to adding gC1, gD1, or gE1 to the tri-HSV-2 vaccine for preventing HSV-1 genital herpes, particularly because adding HSV-1 mRNA immunogens may require lowering the concentrations of HSV-2 immunogens to avoid toxicity [29]. Future studies could evaluate whether adding HSV-1 immunogens not evaluated here may enhance the tri-HSV-2 protection against HSV-1 challenge. 

In mice, T-cells play a vital role in restricting the ability of HSV-1 or HSV-2 to reactivate from latency [30,31,32,33,34]. We previously showed that immunization with tri-HSV-2 mRNA produces strong CD8^+^ T-cell responses to gE2-overlapping peptides [14]. Here, we show that mice immunized with tri-HSV-2 mRNA have robust CD8^+^ T-cell responses to gE1-overlapping peptides. Surprisingly, the responses generated by tri-HSV-2-immunized mice were stronger than by tri-HSV-1-immunized mice as manifested by an increase in polyfunctional IFNγ^+^TNFα^+^IL-2^+^ CD8^+^ T-cells and an increase in CD107a^+^ CD8^+^ T-cells. We also detected a greater CD4^+^ T-cell response to gD1-overlapping peptides in tri-HSV-2-immunized mice than tri-HSV-1-immunized mice. These T-cell responses may translate into the tri-HSV-2 vaccine having an advantage over the tri-HSV-1 vaccine in preventing HSV-1 reactivation from latency. This advantage may be more apparent in the guinea pig genital infection model than the mouse model because recurrent genital lesions and recurrent shedding of HSV DNA can be detected in the guinea pig model [35]. Although antibody responses to gE1 were lower for the tri-HSV-2 than tri-HSV-1 vaccine, the T-cell responses were more robust, suggesting that these opposing results may have offset one another, resulting in comparable protection. The potent CD8^+^ T-cell responses reveal an unexpected benefit of the tri-HSV-2 vaccine against HSV-1 infection. 

Future murine studies to evaluate the protection provided by the tri-HSV-2 vaccine against HSV-1 infection may include using the cornea infection model [36] and encephalitis model [37]. Our current view based on two separate models in mice is that the tri-HSV-1 vaccine offers no clear advantage over the superb protection provided by the tri-HSV-2 vaccine against HSV-1 challenge. The tri-HSV-2 vaccine is currently in human phase 1 safety and immunogenicity trials to prevent genital lesions caused by HSV-2 and potentially HSV-1 (clinical trials identifier: NCT05432583). The results reported here suggest that the tri-HSV-2 vaccine may also be effective at preventing HSV-1 at non-genital sites and may function as a pan-herpes simplex vaccine.

## Data Availability

All data reported in the manuscript will be made available upon request to the corresponding authors.
